# Synchronized locomotion can improve spatial accessibility inside ant colonies

**DOI:** 10.1098/rspb.2023.1805

**Published:** 2023-11-29

**Authors:** Grant Navid Doering, Carmen L. Lee, Kari Dalnoki-Veress

**Affiliations:** ^1^ School of Life Sciences, Arizona State University, Tempe, AZ 85281, USA; ^2^ Department of Physics, North Carolina State University, Raleigh, NC 27695, USA; ^3^ Department of Physics and Astronomy, McMaster University, Hamilton, Ontario, Canada L8S 4K1

**Keywords:** *Leptothorax*, social insects, collective motion, collective animal behaviour, ultradian rhythms

## Abstract

Synchronization is a conspicuous form of collective behaviour that is of crucial importance in numerous biological systems. Ant colonies from the genera *Leptothorax* and *Temnothorax* form small colonies, typically made up of only a few hundred workers, and exhibit a form of synchronized behaviour where workers inside colonies' nests become active together in rhythmic cycles that have a period of approximately 20–200 min. However, it is not currently known if these synchronized rhythms of locomotion confer any functional benefit to colonies. By using a combination of multiple image analysis techniques, we show that inactive *Leptothorax* ants can act as immobile obstacles to moving ants, and that synchronized activity has the potential to reduce the likelihood that individual ants will encounter regions of immobile obstacles that impede access to portions of the nest. We demonstrate qualitatively similar findings using a computational model of confined active particles with oscillating activity.

## 1. Introduction

Synchronized phenomena are pervasive within biology [1,2]. Synchronization also appears in the socially coordinated behaviors of many different organisms. For example, collective oscillations in swarms of bacteria [3], duetting songbirds [4] and human musicians playing in a string quartet [5] are all social contexts that rely on the synchronization of behaviour. Arthropods in particular provide numerous examples of social synchronization, such as fireflies flashing in unison [6], social spiders moving on their web [7,8], groups of honeybees periodically cooling their hive [9] and the chorusing of katydids [10].

**Figure 1.**
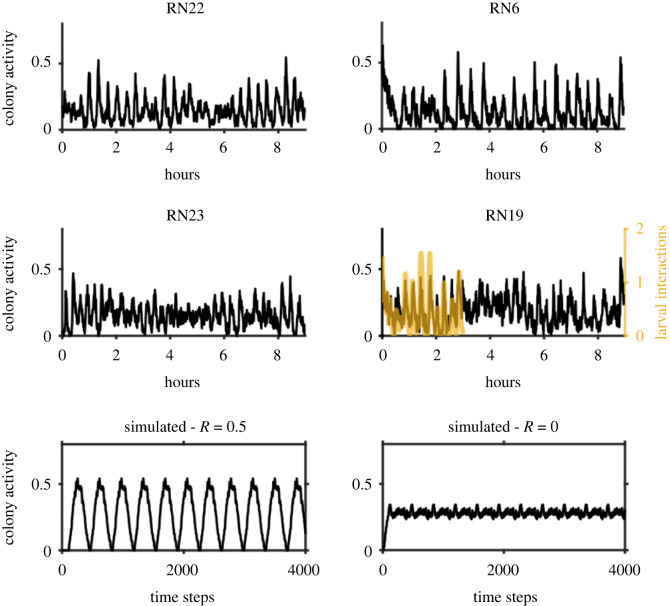
Unfiltered Time series of collective activity (i.e. proportion of individuals moving) in four of the *Leptothorax* colonies analysed in this study. Colony RN6 is an *L. retractus* colony and the others depicted are *L. canadensis* colonies. The plot of colony RN19 includes the 3 h time series of the number of larval interactions that occurred with the 20 focal larvae every 30 s (transparent orange line). The plotted data of larval interactions was smoothed using a 15-point window (i.e. 450 s) Gaussian-weighted moving average. The bottom two plots show example time series of collective activity from simulations of our agent-based model for synchronous agents with *R* = 0.5 and asynchronous agents with *R* = 0. The *y*-axis measures the proportion of agents (*N* = 120 agents) in each simulation that are active at each time step. The other parameters of the model were set to *A* = 100 and *L* = 30 in both simulations.

Among the arthropods, ants rank as one of the most abundant social animal groups on Earth and can exhibit multiple forms of complex coordinated behaviour, such as the collective transport of prey [11], and robust consensus-decision making when choosing a nest [12,13]. Most ant species consist of two morphologically distinct castes: queens and workers [14]. Queens are responsible for reproduction, and workers perform all other tasks for the colony (e.g. gathering food, provisioning brood). One variety of behavioural synchronization in ant colonies that is not yet completely understood is a type of collective activity rhythm that occurs within the nests of ants from several genera (e.g. *Temnothorax*, *Leptothorax*, *Myrmica*, *Solenopsis*) [15–17]. These synchronized activity cycles have been most intensively studied in the twin genera *Temnothorax* and *Leptothorax* [15–21]. *Leptothorax* species form small colonies typically made up of only a few hundred workers [22]. Colonies live in pre-formed cavities (e.g. within rotting sticks, acorns, between rock crevices, etc.; electronic supplementary material, figure S1), which makes it relatively easy to build basic artificial nests for laboratory studies that are structurally similar to natural nests. During collective activity rhythms in *Leptothorax,* most workers become active simultaneously every 20–200 min (depending on the particular colony and species) and begin walking around inside the nest [20,23]. Between these moments of heightened activity, most ants remain motionless (electronic supplementary material, video S1; figure 1). Previous research has shown that these synchronized cycles of activity are generated through physical contact between ants; active ants stimulate inactive ants into moving, and activity spreads like a wave through the colony not unlike an excitable medium [18–20,24].

The benefits that this pattern of locomotion might provide to colonies are still debated [16,25]. The synchronization of rest and activity as well as the rhythmic character of the collective oscillations may have distinct implications for colonies, and multiple hypotheses have been proposed to explain both the rhythmicity [16,23] and synchrony [15,16,26–28] of ant collective activity cycles. One plausible *physical* advantage of synchronization (but not necessarily rhythmicity) is improved spatial accessibility within the nest. Because ants cannot simply walk through each other, inactive ants could act as immobile obstacles that active individuals must either walk around or climb over. Aggregations of immobile obstacles within the nest can therefore be problematic if active ants are attempting to inspect different regions of the nest or access larvae within areas with many inactive individuals. Ants are adept at adjusting their walking speed and trajectories to optimize the flow of workers in nest tunnels and on foraging trails while minimizing instances of jamming [29–31]. However, it is not known if synchronized rhythms of locomotion inside an ant nest might have an analogous effect by reducing the likelihood that individual ants will encounter regions of immobile obstacles that impede access to portions of the nest. In this study, we tested this hypothesis using observations of live *Leptothorax* colonies that exhibited highly rhythmic within-nest activity oscillations. We then built a simple agent-based model of non-interpenetrating mobile oscillators to support our empirical observations.

## 2. Material and methods

### (a) Colony collection and maintenance

Nineteen *Leptothorax* colonies were used in this study (mean no. of ants per colony: 55.8, standard deviation: 36.6; electronic supplementary material, table S1). All colonies were collected from rotting sticks in Royun-Noranda, Quebec in August 2019. Because we were broadly interested in the effects of synchronization on spatial accessibility in this ant genus, we studied all three species belonging to *Leptothorax* that we collected at this site. Out of the nineteen colonies, fourteen of them were *L. canadensis* Provancher 1887, four were *L. retractus* Francoeur 1986, and one colony was the undescribed taxon known as *L*. AF-erg [32]. It should be noted that *Leptothorax canadensis* is a species complex in need of taxonomic revision, but our assessment of the colonies’ morphology suggests that all *L. canadensis* used for this study belong to the undescribed taxon usually referred to as *L*. AF-can [32]. Brood items of various developmental stages were present in each colony, and most of the colonies contained at least one queen (The colonies RN1, RN12, RN14 and RN18 were queen-less). All 19 colonies had their collective activity patterns and properties of their workers' movements analysed automatically using image segmentation and optical flow, and one of the colonies was used for an exploratory analysis of worker–larval interactions during activity cycles (see the remaining sections in the methods for details on these analyses).

Colonies were kept in artificial nests that consisted of a balsa wood slat (2.4 mm thick) with a 38 mm diameter hole drilled through the slat's centre. This hole formed a cavity where the ants could live. Two glass microscope slides (50 × 75 mm) acted as a floor and ceiling for each nest, and an approximately 4 mm nest entrance was cut through the side of each slat, allowing ants to freely leave and return to their nest. Colonies were fed weekly with Spam (Hormel Foods, Minnesota) and honey, and had ad libitum access to water. Before and after being recorded for this study, the ants were maintained in a room at approximately 22C with an artificial light cycle consisting of 12 h of light and 12 h of dark.

### (b) Measuring collective activity

We analysed the spatial patterns in video recordings (each approx. 9 h long) of each of the 19 colonies. We chose to record colonies for nearly 9 h because the periods between activity cycles in *Leptothorax* and *Temnothorax* ants are often shorter than 40 min, so previous studies have able to successfully capture many activity cycles on video by using 9 h recordings [19,20]. All recordings were started in the afternoon between 14.30 and 16.00. We obtained colony-level activity time series of the approximate proportion of ants in a colony that were moving over time (electronic supplementary material, video S1) using the same methods employed in several previous studies [18–20,24]. Our tracking algorithm extracts frames from a video recording every 30 s and then creates binary images by using adaptive thresholding to segment ants inside the nest from the comparatively lighter background. We then compute the number of pixels classified as ants that change from 0 (no ant) to 1 (ant) in each pair of frames and divide this quantity by the total number of pixels detected in the first frame in each pair to estimate the proportion of the colony that has moved between successive frames. Note that pixels that changed from 1 (ant) to 0 (no ant) were ignored to avoid double counting the activity of ants. Tracking collective activity through image thresholding provides a good approximation of activity within the nest although the method's estimates are not immune to noise. For example, if an ant leaves the nest to forage between frames or if an ant climbs on top of another ant, then the total number of ants across a given frame pair will not be identical. Despite these unavoidable limitations, this automated method of tracking collective activity through image binarization can reliably detect when bursts of activity are occurring in a *Leptothorax* nest, and it has been applied in many previous studies [18–21,24,33]. In fact, the period estimates of colonies using this method are in very close agreement to those obtained using the more accurate (but also more invasive, time-consuming and costly [34]) method of tracking individually tagged ants [23].

The dominant period of oscillation for each of the 19 collective activity time series was determined using wavelet analysis. Wavelet analysis allows for the detection of periodic components at multiple points in a time series even if some non-stationarity is present [35]. It is often applied to biological rhythms [35–37], including the ant collective activity oscillations being studied here [19,20,23]. Briefly, we used the continuous wavelet transform function in MATLAB (cwt) to calculate the wavelet magnitudes of different oscillation frequencies at every data point in a time series (for additional details on this method, see the work of Doering and colleagues [19,20]). The oscillation frequency associated with the highest wavelet magnitude (i.e. the rhythm with the strongest signal) was taken as the dominant oscillation frequency of the corresponding time series. The activity time series were smoothed with a Gaussian-weighted moving average filter using a window size of 15 data points (i.e. 450 s) before applying the continuous wavelet transform. Previous work has shown that some smoothing is necessary for an accurate estimation of colony oscillation periods because the cwt function in MATLAB can be sensitive to high-frequency noise [20]. This same work also found that smoothing window lengths between 5 and 15 data points perform nearly identically when estimating a given colony's oscillation period and are suitable for filtering out spurious high-frequency noise. For all other analyses using the activity time series, the raw data (i.e. non-smoothed data) were used.

In addition to within-nest worker activity, we also recorded larval interactions for one arbitrarily selected colony (RN19) by selecting 20 arbitrary larvae and noting every time any ant fed or groomed the chosen larvae over the first 3 h of the colony's video recording. This was done as an exploratory investigation to see if we could find quantitative evidence supporting the anecdotal observation reported for the related ant *Temnothorax allardycei*, where synchronized activity oscillations appear to correlate with times of larval care [18], a vital colony maintenance task. We assessed the synchrony between larval interactions and collective activity level by calculating the cross-correlation between the two time series. The data for larval interactions were smoothed with a Gaussian-weighted moving average filter using a window size of 15 data points (i.e. 450 s) prior to evaluating the cross-correlation.

### (c) Optical flow assessments of inactive ants

**Figure 2.**
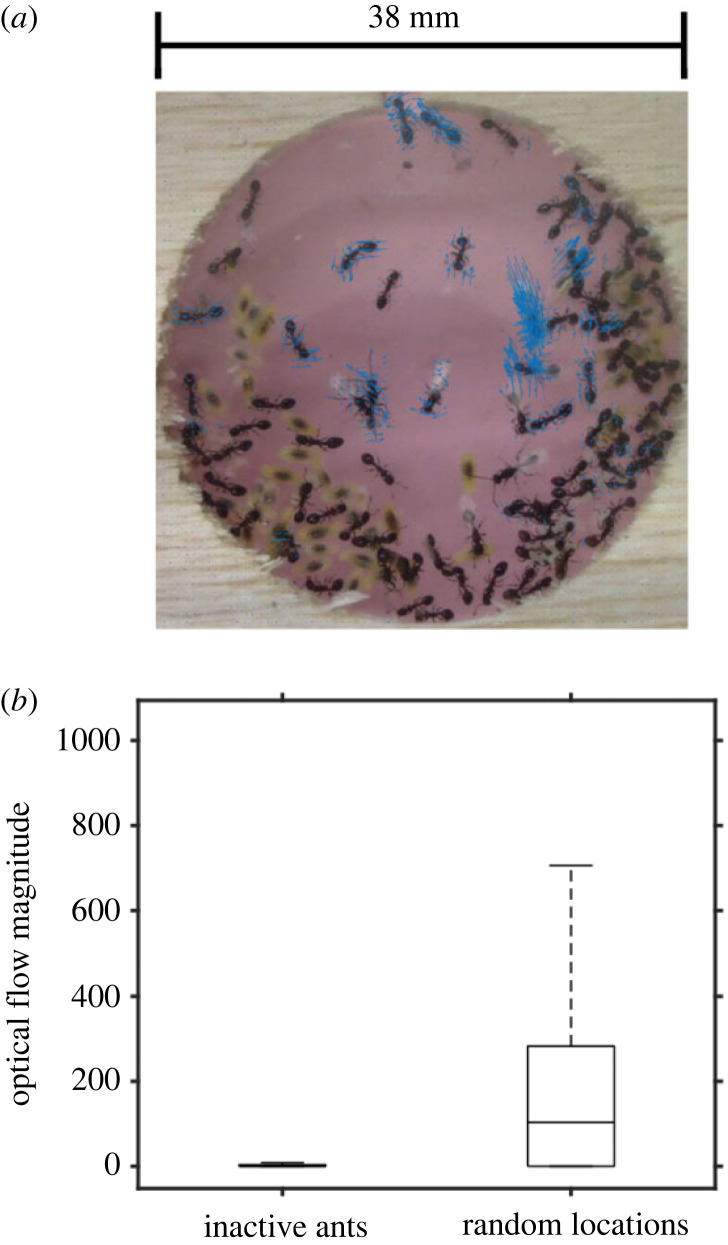
(*a*) The artificial nest of a *L. canadensis* colony (RN23) observed in our study. The yellow oval-shaped objects with dark centres are larvae. The optical flow vectors associated with pixels in this frame have been overlaid on the image. The length of each vector indicates the amount of motion occurring at specific pixels in the current frame compared to the prior frame, and the direction of each vector indicates the direction of the motion. Relatively long blue vector lines can be seen over several ants in the image, indicating that these ants are active (i.e. moving). (*b*) The box plot compares the summed optical flow magnitudes $\left(\sigma_{Px}\right)$ for pixels associated with the locations of inactive ants in the 2.8 min video clip of colony RN23 versus an equal number of randomly sampled pixels. Outlier points have been omitted from the boxplot (but were not omitted from the associated statistical test) for visual clarity.

A visual inspection of our videos gives the strong impression that inactive ants do indeed act as immobile obstacles; ants that are moving tend to walk around inactive nest-mates (electronic supplementary material, video S2). To verify this observation more rigorously, we used optical flow to measure the amount of motion (i.e. displacement vector magnitudes) passing through inactive ants and compared this to the amount of motion at random locations within the nest. The vectors obtained for a pair of images using optical flow correspond to the amount and direction of estimated motion occurring at each pixel. The magnitude of one of these vectors thus conveys the approximate velocity (measured in terms of pixels per frame) of the object (or part of an object) present at the corresponding pixel (figure 2*a*). If active ants do not tend to avoid walking over inactive ants, the average displacement vector magnitudes for pixels that correspond with inactive ant locations should be no different than the average magnitudes of an equal number of randomly chosen pixels.

We used a MATLAB implementation of the Farneback optical flow algorithm [38] (see electronic supplementary material for additional information) to find a displacement magnitude for each pixel in every frame pair from a 2.8 min long segment (at 1 fps) that was haphazardly selected from the 9 h recording of an arbitrarily chosen colony (RN23). The exact duration of this segment (2.8 min) was also chosen arbitrarily, but we specifically avoided using a segment longer than 3 min because this would reduce the number of inactive ants available for analysis. Naturally, the longer a video segment, the less likely it is that a given ant will have remained inactive during the entire segment. The choice to use a frame rate of 1 fps was motivated to avoid large displacements of workers between frames, which would make optical flow analysis more challenging. We filtered out minor fluctuations in optical flow magnitudes between frames that were too small to be associated with moving ants by eliminating optical flow magnitudes that fell below a predefined threshold (1.5 pixels per frame). This threshold value was chosen after inspecting the optical flow vectors associated with a portion of a nest that contained no moving ants. We then evaluated the cumulative sum $\left(\sigma_{Px}\right)$ of each pixel's displacement magnitudes over this 2.8 min segment.

2.1 $$\sigma_{Px}=\sum_{t\;=\;1}^{T}M_{t}\;.$$

Here, *T* denotes the total number of frame pairs from the video segment, and $M_{t}$ is the displacement magnitude of pixel Px at frame pair *t*. This enabled us to estimate the total amount of motion that occurred in each pixel of the video segment over its entire duration. We also automatically segmented all ants that remained inactive over this 2.8 min clip. This automated classification was achieved by counting the number of times each pixel registered a value of 1 across all frames from the 2.8 min clip. We then classified a pixel as being a part of an inactive ant if the pixel was classified as being part of an ant in at least 90% of the frames from the clip. This cutoff was used because only classifying pixels that registered a value of 1 in every frame would undercount inactive ants. For example, if the image binarization happened to not detect a particular inactive individual in just one of the frames of the video segment, then that ant would be incorrectly classified as not being inactive. There were a few instances where only part of an ant moved during the video clip, so to filter noise and improve our detection of inactive ants, we removed small clumps of pixels that were classified as inactive but were too small to be inactive ants. This filtering was accomplished automatically by setting a threshold to remove any connected sets of pixels that contained fewer pixels than which usually constituted the smallest ants within a colony. Finally, we compared the $\sigma_{Px}$ values of pixels classified as comprising inactive ants with an equal number of randomly chosen pixels.

To evaluate whether the optical flow results obtained for the single clip analysed from RN23 were robust, we also performed the same analysis on 1 min segments from all 19 colonies; In each colony the $\sigma_{Px}$ values of inactive-ant pixels were compared to the $\sigma_{Px}$ values of an equal sample of random pixels. For each colony, we identified the start points, peak points, and end points of the collective cycles in a colony's activity record using the *findpeaks* and *gnegate* functions in MATLAB (electronic supplementary material, figure S2), and then we arbitrarily chose a single collective activity cycle to subject to optical flow analysis. The starting frame for the 1 min segment within each colony's chosen activity cycle was set as the frame corresponding to 30 sec before the midpoint of the cycle. Additionally, in order to assess whether our results would be consistent over an entire 9 h colony recording, we arbitrarily selected colony RN22 and gathered a set of twenty 1 min segments from this colony's recording whose locations in the time series were chosen using a pseudorandom number generator (electronic supplementary material, figure S3). We then conducted the same optical flow analysis on each of these 20 segments. Because the total number of frames from colony RN22 that were analysed with optical flow exceeded the number of frames that were analysed from colony RN23, we reduced the resolution of the frames of RN22 prior to analysis to speed up the calculation of the optical flow magnitudes. This reduction in resolution to obtain faster image processing times was likewise performed for all other image-based analyses of the colony recordings; the only analysis that retained the original resolution was the preliminary optical flow analysis of colony RN23. Because the ants were darker than the background in our images, ants could still be segmented automatically regardless of this reduction in resolution.

### (d) Quantifying spatial inaccessibility

To define a simple metric to quantify spatial inaccessibility, we measured the number of inactive ants within the largest aggregation of inactive ants inside the nest at each moment of the activity time-series. We refer to this metric as the maximum local density (*MLD*), and we calculated time series of *MLD* for all 19 colonies' activity recordings. Specifically, we partitioned each video frame into a regular grid with 16 equally sized sectors that each consisted of 68 × 68 pixels (approximately 9.7 × 9.7 mm) and counted the number of pixels every 30 s that made up the inactive ants (i.e. the segmented pixels that did not change between a pair of successive frames) in each sector. The number of such pixels in each sector was then divided by the approximate number of pixels that typically made up the entire body area of a single ant (62 pixels in the case of *L. retractus* and 80 pixels for both *L. canadensis* and *L*. AF-erg) to estimate the number of individuals in every sector. The number of inactive ants in the sector with the most inactive ants was then used as the value of *MLD* in the nest for that time point. Thus, for each data point in a colony's activity time series (i.e. for each 30 sec interval in a colony's activity recording) we were able to obtain a *MLD* value as well. This method of determining local density is similar to those applied to other insect aggregations [39].

Importantly, this metric is not trivially guaranteed to vary with colony activity level. For example, even if most ants are moving during an activity burst, the ants that do remain inactive could conceivably still be concentrated in a dense aggregate. Alternatively, if a nest was sparsely populated and ants lacked a tendency to aggregate when inactive, then one would likewise not observe a correlation between activity level and *MLD*. Because a low value of *MLD* necessarily indicates that *all* regions of the nest contain few inactive ants, a negative relationship between activity and *MLD* would thus entail a benefit to spatial accessibility from synchrony because when more of the colony is active, there will be fewer immobile obstacles throughout the entire nest. Moreover, if MLD is low, any region that that an active ant visits will only contain a small total number of inactive ants. We also computed a proportional variant of our *MLD* metric (pMLD) by dividing every *MLD* value from a given colony by the number of adult ants in that colony.

### (e) Spatial accessibility in and around brood piles

We used a targeted application of optical flow analysis on aggregations of inactive ants that were resting on brood piles in all 19 colonies. This was done to more directly test the notion that inactive ants can limit the amount of available space that active ants are able to easily access. To do this, we took the activity cycles that were chosen for the 1 min window optical flow analyses described above and divided them into multiple segments that were each 30 s long. If regions of inactive ants are in fact less accessible to active ants, we would expect that the proportion of space that active ants are patrolling at any particular time would be negatively and linearly correlated with the number of inactive ants in and around the brood pile. Conversely, if ants could travel to any area of the brood pile that they wanted regardless of the presence of inactive ants, then we would not generally expect such a relationship because just one or two active ants would be able to walk over many parts of the brood pile. An additional prediction of the hypothesis that inactive ants physically block the motion of active ants is that optical flow magnitudes should differ significantly between areas with inactive ants and the areas immediately next to inactive ants because any active ants that walk up to areas with inactive ants will be prevented from proceeding farther into that area of the brood pile.

To quantitatively test these two predictions in each colony, we first cropped the frames of the recordings to focus on a colony's brood pile (or a single arbitrarily chosen brood pile in cases where a colony had more than one) and classified the pixels in these brood piles that comprised inactive ants during each 30 second window. The classification of inactive ants for optical flow purposes was performed in the same way as already described earlier in this section (i.e. pixels that were classified as containing an ant for more than 90% of the frames in a given time interval). We ensured that our brood pile crops were consistent in their size across all colonies, being 100 × 150 pixels (or 150 × 100 pixels in cases where the brood pile had a more horizontal orientation). Each brood pile was then divided into a regular grid consisting of 150, 10 × 10 pixel sized regions. For each 30 s interval, we counted the total number of detected inactive ants in the brood pile along with the proportion of regions (i.e. out of the 150 regions) in the brood pile that contained any activity (i.e. non-zero optical flow magnitudes) during that 30 sec interval. Finally, for each 30-second interval, the summed optical flow magnitudes for all pixels associated with inactive ants was compared with the summed optical flow magnitudes for all pixels that were situated immediately adjacent to the inactive ants. The pixels that were immediately adjacent to the inactive ants were determined in an unbiased, automated way using morphological dilation (implemented with the MATLAB function *imdilate*). This operation increases the size (in terms of pixels) of the segmented ants but preserves their approximate shape. Essentially, a ring approximately 10 pixels thick (i.e. a square structuring element of value 10) was added around each inactive ant, and the pixels that comprised this added area were defined as being immediately adjacent to the inactive ants.

### (f) Mobile oscillator model

We designed a simple mobile phase oscillator model to validate and generalize our empirical observations. Our principal goal with this model was to test if a simple system of synchronous, non-interpenetrating agents that were confined at a density approximating real colonies would exhibit qualitatively similar improvements in our spatial inaccessibility metric *MLD*. This discrete-time model consisted of a square, continuous 2D space of size *L* × *L* (arbitrary length units) where a population of *N* non-interpenetrating agents (circular particles with a diameter of 1 unit) could move. Each agent could be in either one of two states at any one time: active or inactive. When inactive, an agent would remain motionless at its current coordinates. When active, agents would perform a correlated random walk through the simulation space (see details in next paragraph). Each agent transitioned between being active and inactive by following an internal oscillator whose phase spanned 0–360°. The phase of each agent's oscillator would advance by one degree at each time-step of a simulation and loop back around to 0° each time it reached 360^°^. All agents were always made to start in the inactive state at the beginning of a simulation. The parameter *A* controlled the proportion of time that ants spent active versus inactive. Whenever an agent's phase passed 350° it would enter the active state and remain active until its phase progressed by *A* degrees, at which point it would enter the inactive state. After its first activation, a simulated agent would therefore repeatedly cycle between the active state (each time lasting for *A* + 1 time steps) and the inactive state (each time lasting for 360-*A* + 1 time steps).

At every time-step that an agent spent active it would undergo a correlated random-walk, meaning that it would independently decide on the direction of its next movement by randomly selecting a heading that was within 45° of its current heading and then proceed to walk 1 unit of distance in the chosen direction. Agents were prohibited from spatially overlapping with other agents (i.e. they could not interpenetrate). If an active agent attempted to make an invalid movement during its random walk (this would either be a movement that would result in overlap with another agent or cause it to step outside the bounds of the simulation arena), it would randomly select a new heading and attempt to walk in that direction.

We quantified the level of phase synchrony between agents using the Kuramoto order parameter *R* [40].

2.2 $$R\;=\;\left|\frac{1}{N}\overset{N}{\underset{\;j=1}{\sum }}e^{i\vartheta_{j}}\right|.$$

In equation (2.2), *N* represents the number of oscillators (i.e. ants/agents), and $\vartheta_{j}$ represents the phase of oscillator *j*, where j ∈{1…N}. The order parameter ranges from 0 (no phase synchrony) to 1 (complete phase synchrony). This modelling approach allowed us to study the effects of synchrony on spatial inaccessibility in general while being agnostic to both the underlying mechanism the agents use to synchronize and any effects of social interactions between agents. We measured the spatial inaccessibility of simulated nests using the same *MLD* metric that we defined for the empirical data.

We set our model's parameters to roughly approximate the conditions seen in our artificial nests to see if the empirically observed negative relation between *MLD* and activity could be recreated in a highly simplified setting. We simulated an arena with 120 agents, set the activity level of individuals to be *A* = 100, set the size of the arena to be *L* = 30, and set the synchrony of the agents to be *R* = 0.5. This combination of parameters settings produces collective activity patterns that resemble those seen in real colonies (figure 1). To set a desired level of synchrony between the oscillators in the model, we randomly assigned phases to each oscillator at the start of a simulation (but before the simulation was allowed to proceed) until the resulting value of *R* was within 0.01 of the desired value (in the case of the present study: *R* = 0.5). The decision to set R to 0.5 was motivated by the fact that this produces collective oscillations with amplitudes close to those seen in our real colonies. Because *Temnothorax* and *Leptothorax* workers inside the nest often spend the majority of their time resting and inactive [41,42], we selected a value of the parameter A so that this would also be true for the agents in our model. By setting *A* = 100, individual agents in the model would spend (360–101)/360 = 71.9% of their time inactive.

There will be a periodic structure to the collective activity in our model (figure 1) regardless of the value of *R*. This is because the activation of individual ants follows a periodic pattern, and the sum of any group of periodic signals will almost always be periodic. For example, consider the case of two sinusoidal oscillators. If the order parameter is *R* = 0, then the two signals will be perfectly anti-phase and the sum of the two signals will cancel out and the resulting collective signal will be a flat line. However, if the order parameter is anything other than exactly zero, then the sum of the two signals will have a detectable periodicity because the phase difference between the two signals will remain constant. Likewise, the total number of active agents over time will thus also be periodic even if synchrony is set to a low value because the internal clocks of individual agents in our model are completely periodic. Despite this periodicity, the motion of ants in the simulation area was stochastic, so the time series of the *MLD* metric in our model were not deterministic.

Density, which was defined as the number of agents divided by the area of the simulation arena $D_{a}=N/L^{2}$, was set to $120/30^{2}\;=0.133$ agents per length units squared. This level of density is similar to that of real colonies from this study. The approximate body dimensions of individuals from our *L. canadensis* colonies are approximately 3.0 × 0.4 mm (see published morphology data on *L. athabasca* for detailed measurements on a closely related North American species with a similar body size [43]) and the diameter of an agent in our model is 1 unit. Thus, if we assume that 1 unit in the model is equal to approximately 1 mm of real distance then this results in the surface area of individual agents $\left(\pi *0.5^{2}=0.79\;{\mathrm{mm}}^{2}\right)$ being roughly similar to the area of real individual ants $\left(3.0*0.4=\;1.2\;{\mathrm{mm}}^{2}\right)$. If we calculate nest density the same way for real ants as we do for the model, then the density of a colony of 120 ants in one of our circular artificial nests (19 mm radius) is $120/(\pi *19^{2})\;=0.11$ ants per millimetre squared, which is close to the model's value of 0.133 agents per length units (i.e. millimetres) squared. Lastly, we divided the simulation arena into 16 sectors to calculate the *MLD* metric for our simulation output.

## 3. Results

The recorded colonies exhibited synchronized activity cycles (figure 1; electronic supplementary material, figure S4) with a period generally near 20 min (*L. canadensis* (*N* = 14): 22.9 ± 9.9 min; *L. retractus* (*N* = 4): 21.8 ± 3.7 min; *L*. AF-erg (*N* = 1): 15 min). Larval interactions in colony RN19 were also cyclic and synchronized with locomotor activity level (figure 1; maximum cross-correlation at zero lag: *r* = 0.754, *p* < 0.00001). The mean displacement magnitudes determined through optical flow for the 2.8 min clip of colony RN23 were significantly lower for pixels associated with inactive ants (figure 2; Wilcoxon rank sum test: *W* = 48974000, *p* < 0.00001), indicating that active ants do maneuver around inactive ants. This was further verified in our analysis of colony RN22; the displacement magnitudes occurring over inactive ants were significantly less (Bonferroni corrected *α* = 0.05/20 = 0.0025) then the displacement magnitudes at random pixels at all 20 sampled segments of the time series (electronic supplementary material, table S2; *p* < 0.00001 for all cases). Applying this optical flow analysis across all 19 colonies confirms that active ants generally tend to avoid walking over inactive ants. For every one of the 1 min windows extracted from the arbitrarily selected activity cycle of each of the 19 colonies, the optical flow magnitudes associated with inactive ants were lower than the optical flow magnitudes associated with random locations, and these differences were statistically significant in 17 out of the 19 colonies (electronic supplementary material, table S3; Wilcoxon rank sum test: *p* < *α* = 0.05/19 = 0.0026).

**Figure 3.**
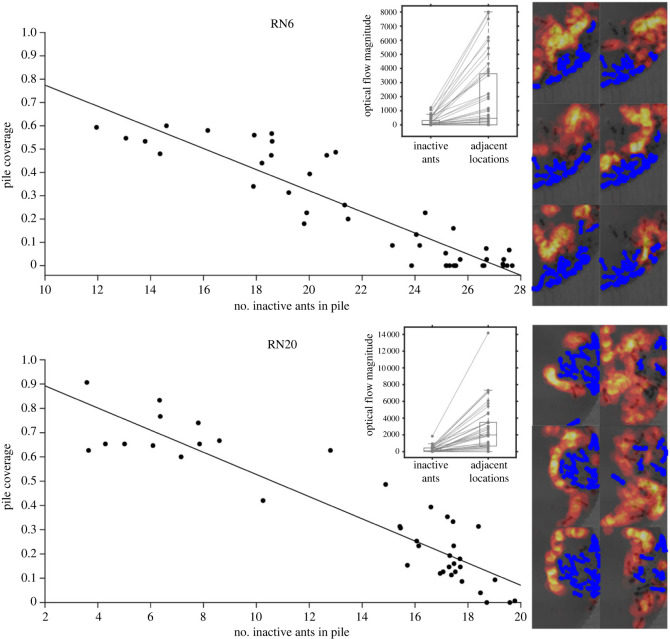
The relationship between the approximate number of inactive ants in the colony's brood pile and the proportion of regions in the brood pile (out of 150 regions total) where activity was detected with optical flow analysis (referred to here as pile coverage). The data from two representative colonies (RN6 and RN20) are plotted. Each data point represents a single 30 s window from the chosen activity cycle of the plotted colony. Optical flow visualizations for the brood piles of these two colonies during different 30 s windows are also shown (six images per colony are presented). In each image, brighter red areas represent relatively more activity. Pixels that were automatically classified as belonging to ants that were inactive during the entire 30 s interval are highlighted in blue. The box plot insets in each panel depict the summed optical flow magnitudes associated with inactive ants alongside the summed optical flow magnitudes for pixels that were immediately adjacent to inactive ants. Each line joining paired observations represents the data from one of the 30 s time windows during the respective colony's chosen activity cycle.

Analysing the ants around brood piles from all 19 colonies reinforces our finding that concentrations of inactive ants might act as barriers that impede active ants from entering certain areas. Visualizations of optical flow magnitudes around the brood piles from multiple colonies clearly reveal that dense concentrations of inactive ants have the potential to act as barriers that active ants cannot easily penetrate (figure 3). The approximate number of inactive ants in a brood pile at a given time was significantly (*α* = 0.05/19 = 0.0026) and negatively correlated with the proportion of regions in a brood pile containing activity in 18 out of the 19 colonies (figure 3; electronic supplementary material, figure S5 and table S1; *p* < 0.0001 for all cases except colony RN18 [*r* = −0.402, *p* = 0.0091]). Furthermore, activity on top of inactive ants in the brood piles was significantly lower than activity immediately adjacent to inactive ants for 18 out of the 19 colonies (figure 3; electronic supplementary material, figure S6; table S1; Wilcoxon signed-rank test: *p* < 0.0001 for all but two cases, and only colony RN18 was non-significant [*p* = 0.0225]). Thus, even when there are many ants active during a given interval of time, active ants tend to not walk over areas with ants that remained inactive during that interval. By contrast, active ants do walk through regions that *previously* contained inactive ants (figure 3).

**Figure 4.**
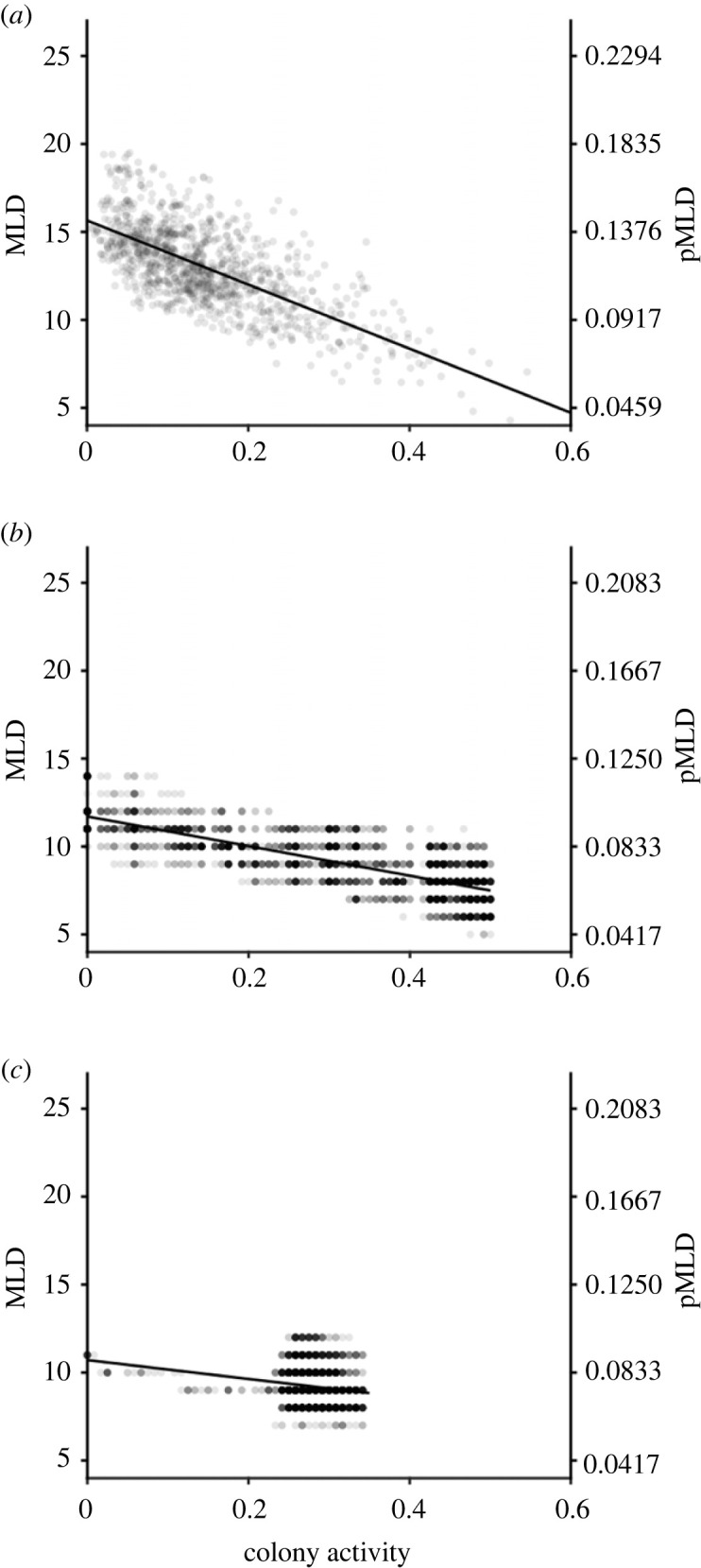
The relationship between colony activity and the spatial inaccessibility metric max local density (*MLD* and its proportional version *pMLD*) for colony RN22 (*a*), for a 4000 time-step simulation of our agent-based model where *R* = 0.5 (*b*), and for a 4000 time-step simulation of our agent-based model where *R* = 0 (*c*). The translucent grey dots depict the raw data points, and the solid black line in each panel is the least-squares fit of the data. The reason that there is still a long negative slope when *R* = 0 is due to the fact that the initial conditions of the model always had all agents start in the inactive state, which resulted in a transient portion at the beginning of the time series with high *MLD* values.

We found that our spatial inaccessibility metric *MLD* (maximum local density) was significantly negatively correlated with colony-level activity in all 19 colonies (*p* < 0.0001 for all cases; figure 4*a*; electronic supplementary material, table S1 and figure S7). These analyses demonstrate that during activity peaks, activity generally propagates through all the regions of a nest that previously contained inactive ants. The negative relationship between activity and *MLD* also does not depend on specific choices regarding the number of regions we used in calculating *MLD* (electronic supplementary material, figure S8). The correlation strength of *MLD* versus activity did not depend on the number of ants in a colony (Pearson correlation: *r* = 0.288, *p* = 0.2318). Also, even though our simple model ignores many nuances of how ants interact or how such interactions might alter rest/activity patterns, the behaviour of the model qualitatively matches that seen in real colonies; *MLD* is reduced at higher activity levels (Simulation 1 (*R* = 0.5), Pearson correlation: *r* = −0.832, *p* < 0.0001; figure 4*b*; Simulation 2 (*R* = 0.5), Pearson correlation: *r* = −0.853, *p* < 0.0001; Simulation 3 (*R* = 0), Pearson correlation: *r* = −0.198, *p* < 0.0001; figure 4*c*). Additionally, when the agents in the model are asynchronous (i.e. when *R* = 0), the values of *MLD* occupy a narrow range and fail to reach the lower values that appear when the agents are synchronous (i.e. when *R* = 0.5) (figure 4*c*).

## 4. Discussion

These results show that synchronizing moments of activity and rest can lead to better spatial accessibility inside ant nests. Inactive ants behave as immobile obstacles that constrain the potential locations where active ants can walk. The synchronization of worker activity thereby makes it possible for ants to be active in more regions of the nest as more ants become active. The results of our model suggest that this effect arises even in highly simplified conditions. Furthermore, every colony across the three species used in this study showed a negative relationship between activity and the *MLD* spatial inaccessibility metric, which implies that this phenomenon is common in *Leptothorax*.

Synchronous active-rest oscillations may be present in other social situations that share some of the same characteristics as ant nest cavities, such as bacteria colonies, other social insects, or gregarious vertebrates restricted to living in small habitat patches. In such cases, synchrony might similarly improve spatial accessibility. For instance, our results resemble the coordinated motions observed in huddles of emperor penguins; these birds stand in tight formations and intermittently become active together to allow individuals at the periphery of the huddle to access the crowd's warmer interior [44]. The simultaneous flashing of fireflies in mating swarms also appears to have a somewhat analogous benefit. Asynchronous flashing would cause the flash patterns of males to interfere with each other, reducing visibility for females seeking mates [45]. Instead of clearing visual ‘clutter’ in the case of fireflies, synchrony inside *Leptothorax* nests reduces the spatial clutter caused by immobile ants.

Several other hypotheses have previously been proposed for ant activity cycles, including faster task completion [26], energy conservation [15,46], rapid information transfer [47,48] and control over foraging rates [27]. These hypotheses are not mutually exclusive (either with each other or with our findings) and collective activity cycles may confer all of these benefits. Our work demonstrates an effect of synchronization on the potential mobility of ants in the nest, but this effect does not depend on the collective activity oscillations being rhythmic. The fact that *Leptothorax* activity cycles tend to be periodic may have functional implications for colonies that are distinct from synchronization. It has, for example, been hypothesized that periodic cycles could facilitate a faster flow of information within a nest, but a recent study found data contrary to this idea [23]. Although our work is the first experimental demonstration that synchronization has spatial implications for where *Leptothorax* ants are free to be active inside the nest, one limitation is that it is not yet evident whether improved spatial accessibility inside the nest has any fitness implications for colonies. Indeed, it has also been proposed that synchrony may conceivably have negative impacts on colony fitness [18]. Regardless of whether spatial accessibility is improved through synchronization, synchronous activity may still result in a net reduction of worker efficiency when completing tasks if active ants interfere with each other's behaviour.

Nevertheless, there are at least two ways that better spatial accessibility could potentially benefit colony fitness. One potential fitness advantage of high spatial accessibility is efficient brood tending. The logic is that because inactive ants can congregate near larvae (see for example the concentration of inactive ants next to brood items in figure 2*a*), poor accessibility of these regions could interfere with the ability of workers to interact with brood. This, in turn, could lead to less frequent feeding and grooming, harming eclosion rates. Other lines of evidence point in this direction. For example, collective activity cycles are stronger near the brood pile [24]. The coherence of colony oscillations are also weaker when just pupae (which do not require frequent tending) are present or when a colony has no brood at all [49]. Another form of rhythmic activity in ants (circadian rhythms) is also affected by the presence of brood [50].

The hypothesis that better spatial accessibility might assist with brood care is complementary to a previously proposed theoretical model which found that active ants might mutually exclude one another from feeding the same larvae during synchronized cycles [28]. The argument is that if more than one ant cannot feed the same brood item at the same time, then if ants awaken simultaneously, they would be less likely to redundantly feed the same brood item multiple times in a short period of time. Because provisioning larvae with food is not the only type of brood-worker interaction, the sustained presence of many inactive ants that would be found in an asynchronous colony might analogously impair other forms of brood-worker interactions. For example, *Leptothorax* and *Temnothorax* ants must also groom the larvae, which helps prevent fungal infections [51], and the labial secretions of larvae form part of the diet of workers [52,53]. If individual workers need to feed off of larval secretions frequently, then ants in an asynchronous colony would be most likely to be active at times when few larval items are accessible to feed on because, as we have shown in the present study, inactive ants can block active ants. Any larval items with inactive ants resting on them would thus be more inaccessible to an active ant looking to feed. The correlation that we observed between overall colony activity and larval interactions is consistent with this hypothesis that better spatial access in general might benefit access to brood as most worker-brood interactions seem to occur when spatial access is maximized. However, this observed correlation must be considered tentative at this point since we only performed an exploratory analysis on a single colony. If such a link turns out to be robust, it could have implications for whether or not better spatial accessibility might have ultimate fitness implications. Whether or not the general improved spatial accessibility identified in this study translates to easier brood access for workers is thus a promising area for additional research.

A second possible benefit of spatial accessibility also relates to brood access. Like workers, queens of some myrmicine ants (including *Temnothorax*) are known to visit multiple larvae during activity bouts and feed off of their labial secretions [52,54]. Queens can also trigger large activity waves [19] and this appears to clear a path for the queen and gives her improved access to larval secretions [52]. If inactive ants cluster over larvae, having a colony with asynchronous activity would therefore mean that a colony's queen would most often be active when much of the larvae are covered by aggregations of inactive ants. Easier access to larval secretions for a colony's queen could potentially thereby lead to better nutrition and higher fecundity [54].

Even though the fitness implications of synchrony remain unknown, our work demonstrates (1) that inactive ants can act as locally clogged regions that dictate where active ants can freely move, and (2) that synchronization reduces the density of inactive ants throughout the nest, thus increasing the number of possible locations where ants can visit within their nests at a given time. Our findings will also have relevance regardless of the exact spatial distribution of ants in a nest; because inactive ants behave as *immobile* obstacles, the total number of inaccessible locations in a nest will shrink as more ants awaken. However, future work that examines how ants' collective activity patterns might depend on their nest geometry would be useful to address how ants successfully manage to tend brood in increasingly cramped spaces and whether or not synchrony has fitness implications for colonies. Indeed, natural nests of *Leptothorax* and *Temnothorax* are capable of being even more densely populated than the artificial nests used in this study (electronic supplementary material, figure S1). More detailed modelling work that considers the effects of nest geometry could also be beneficial. For example, the slope of MLD versus activity in our current model is shallower than the slope seen in colony RN22, which could be due to our simplified assumptions about how agents are distributed in the simulation space. Species that typically differ in their nests**’** geometry (e.g. narrow tubular sticks versus flat rock crevices) may also be promising targets for comparative work. It is known from models of insect synchronization that periodic group oscillations can emerge from individuals that lack an intrinsic periodicity [20,55–57]. The social processes that affect the emergence of collective behaviour in these contrasting situations may plausibly differ as well, though this still needs to be investigated. Fortunately, due to the ease with which individuals and entire colonies can be manipulated and observed, *Leptothorax* ants are a useful taxon for studying both synchronization in social systems and collective motion in cramped spaces.

## Data Availability

All image sequences and empirical data used in this study, the code for our agent-based model, and the code used for our statistical, time-series and image analyses are available and archived on Zenodo (https://doi.org/10.5281/zenodo.10042488) [58]. Supplementary material is available online [59].
